# Expression of Concern: MicroRNA-96-5p promotes proliferation, invasion and EMT of oral carcinoma cells by directly targeting FOXF2

**DOI:** 10.1242/bio.059509

**Published:** 2022-07-01

**Authors:** Haiyan Wang, Ning Ma, Wenyue Li, Zuomin Wang

Concerns have been raised about *Biology Open* (2020) **9**, bio049478 (doi:10.1242/bio.049478), including the potential fraudulent use of author details. We will update this notice as soon as possible.

